# Supplementary material for: TAZ links exercise to mitochondrial biogenesis via mitochondrial transcription factor A
Source: Nat Commun. 2022 Feb 3;13:653. doi: 10.1038/s41467-022-28247-2 (PMC8814203; doi:10.1038/s41467-022-28247-2)
Supplement: Supplementary file 1 — Supplementary Information [file 41467_2022_28247_MOESM1_ESM.pdf]

Supplementary Information

TAZ links exercise to mitochondrial biogenesis via mitochondrial transcription factor A

Jun-Ha Hwang[1], Kyung Min Kim[1], Ho Taek Oh[1], Gi Don Yoo[1], Mi Gyeong Jeong[2], Hyun Lee[1], Joori Park[1], Kwon Jeong[1], Yoon Ki Kim[1], Young-Gyu Ko[1], Eun Sook Hwang[2,*], Jeong-Ho Hong[1,*]

[1]Department of Life Sciences, School of Life Sciences and Biotechnology, Korea University, Seoul 02841, Korea

[2]College of Pharmacy, Ewha Womans University, Seoul 03760, Korea

*Correspondence: jh_hong@korea.ac.kr (J.H. Hong) or eshwang@ewha.ac.kr (E.S.H.)

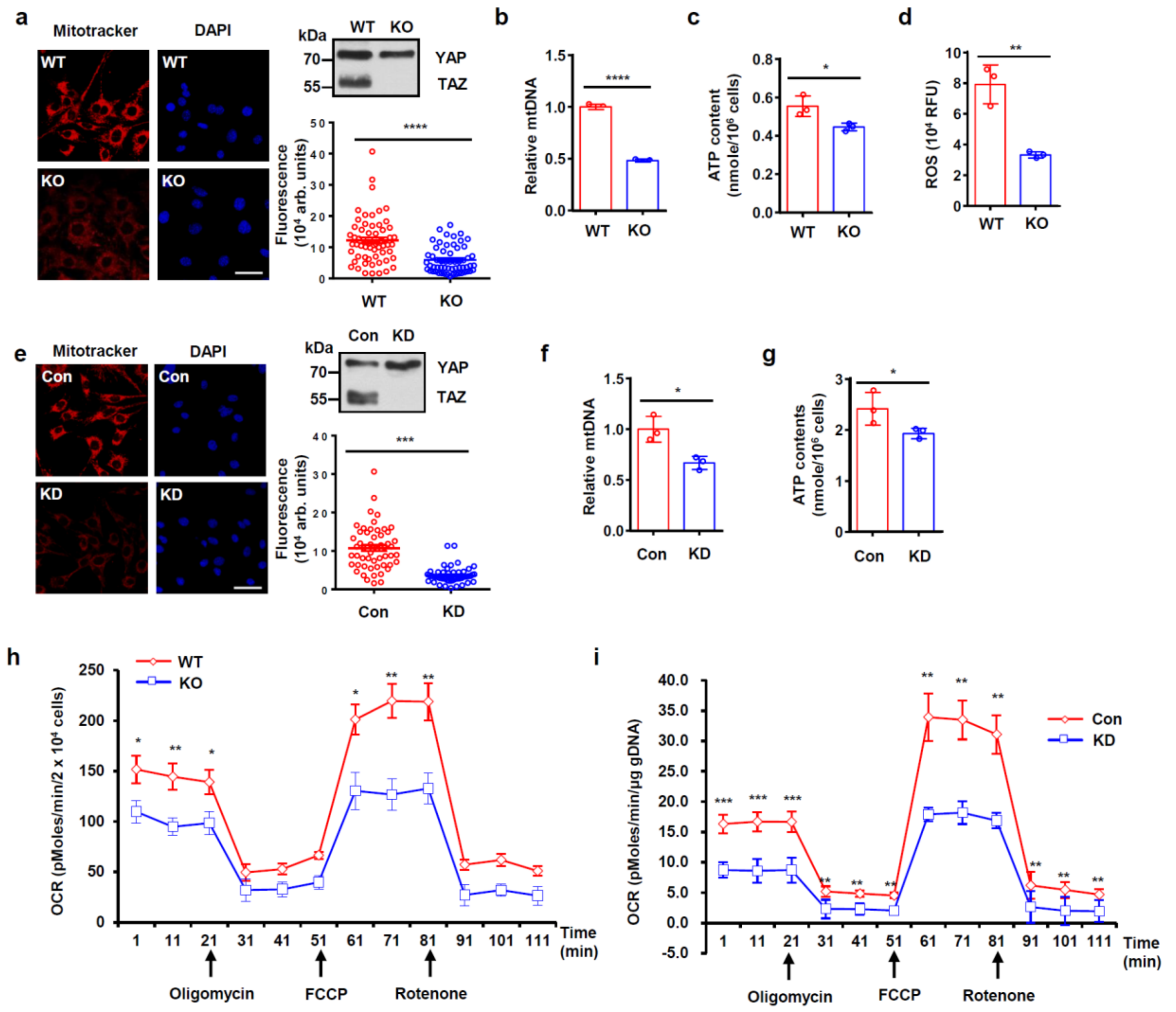

Supplementary Fig. 1 TAZ upregulates mitochondrial mass and oxygen consumption rates *in vitro*. a) Wild-type (WT) and TAZ knockout (KO) MEF were stained with MitoTracker to detect mitochondria. Cell nuclei were counterstained with DAPI. Scale bar = 50 µm. The fluorescence intensity of each single cells was measured using ImageJ software and values are presented as arbitrary units (n = 62 for WT and n = 58 for KO, **** $p < 0.0001$). TAZ knockout was verified via immunoblotting (upper right). **b**) Genomic DNA was isolated from WT and TAZ KO MEF. The relative mitochondrial DNA content was determined via quantitative PCR (qPCR) using mitochondria-encoded *Cox2*-specific primers. Data were normalized via qPCR with primers for nuclear-encoded *β-globin*. The experiment was performed in triplicate (**** $p < 0.0001$). **c**) Cellular ATP content in WT and TAZ KO MEF was determined via ATP bioluminescence assay. The experiment was also performed in triplicate (* $p = 0.0305$). **d**) The production of reactive oxygen species (ROS) in WT and TAZ KO MEF was analysed. The mass of ROS is shown as relative fluorescence units

(RFUs) (** $p = 0.0035$). **e**) Control (Con) and TAZ knockdown (KD) C2C12 myoblasts were stained with MitoTracker to visualise mitochondria. Cell nuclei were counterstained with DAPI. Scale bar = 50 μm. The fluorescence intensity of each cell was determined using ImageJ software and data are shown as arbitrary units (n = 53 for WT and n = 51 for KO, **** $p < 0.0001$). TAZ knockdown was confirmed via immunoblotting (upper right). **f**) Genomic DNA from Con and TAZ KD C2C12 myoblasts was analysed via qPCR to quantify the relative abundance of mitochondrial DNA. The experiment was performed in triplicate (* $p = 0.0155$). **g**) The cellular ATP levels of Con and TAZ KD C2C12 myoblasts were quantified via ATP bioluminescence assay. The experiment was performed in triplicate (* $p = 0.0339$). **h**) The cellular oxygen consumption rate (OCR) of WT and TAZ KO MEF was measured using an XF analyser following treatment with 1 μg/mL oligomycin, 0.5 μM carbonyl cyanide 4-(trifluoromethoxy) phenylhydrazone (FCCP), and 0.1 μM rotenone. The experiment was performed in triplicate (1 min; * $p = 0.0262$, 11 min; ** $p = 0.0096$, 21 min; * $p = 0.0253$, 61 min; * $p = 0.012$, 71 min; ** $p = 0.0032$, 81 min; ** $p = 0.0057$). **i**) The cellular oxygen consumption of Con and TAZ KD C2C12 myotubes was measured following treatment with 1 μg/mL oligomycin, 0.5 μM FCCP, and 0.1 μM rotenone using an XF24 analyser. The experiment was performed in triplicate (1 min; *** $p = 0.0008$, 11 min; *** $p = 0.0007$, 21 min; * $p = 0.0009$, 31 min; ** $p = 0.0015$, 41 min; ** $p = 0.0026$, 51 min; ** $p = 0.0015$, 61 min; ** $p = 0.0084$, 71 min; ** $p = 0.0069$. 81 min; ** $p = 0.0062$, 91 min; ** $p = 0.0077$, 101 min; ** $p = 0.0018$, 111 min; ** $p = 0.003$). Data are presented as mean ± SEM for panel **a** and **e**, ± SD for panel **b**, **c**, **d**, **f**, **g**, **h**, and **i**. Statistical significance was analysed via two-sided *t*-test for panel **a**, **b**, **c**, **d**, **e**, **f** and one-side *t*-test for panel **g**, **h**, **i**. Source data are provided as a Source Data file.

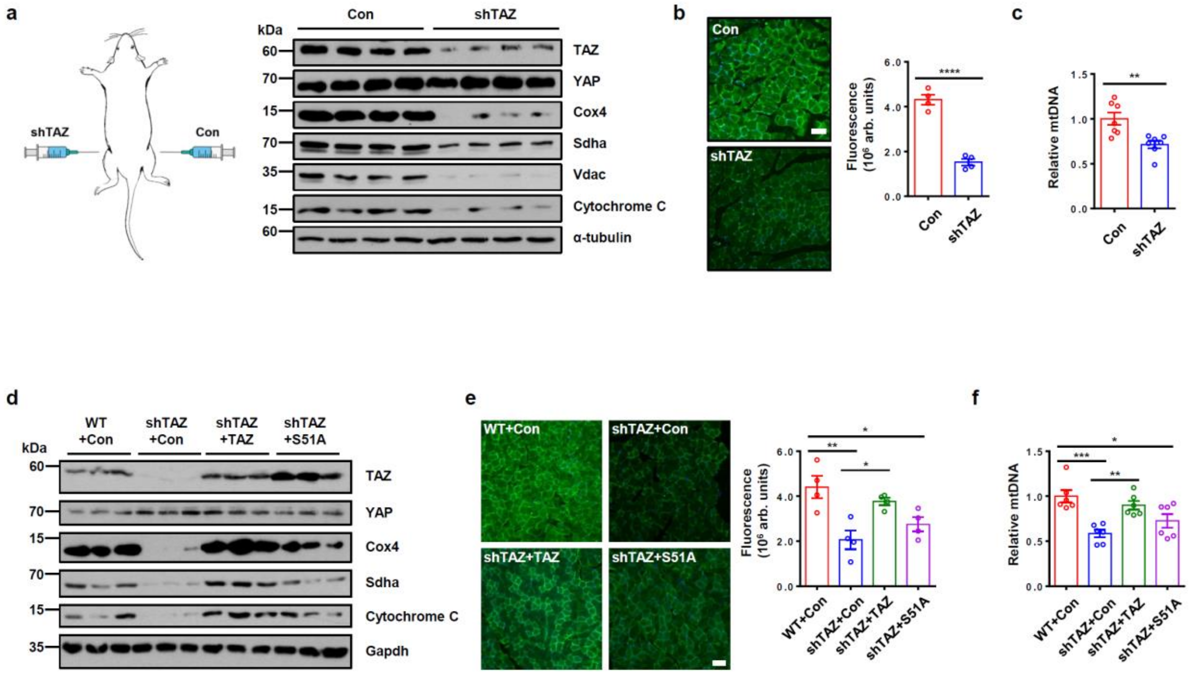

Supplementary Fig. 2. Local depletion of TAZ in gastrocnemius muscle of adult mouse decreased mitochondrial mass. a) Mouse gastrocnemius muscle was transduced with control (Con) or TAZ shRNA-containing (shTAZ) AAV6 virus to locally deplete TAZ in muscle tissue. To exclude individual variation in mouse activity, Con or shTAZ AAV6 virus were injected into the right or left gastrocnemius muscle of the same mouse respectively. Protein isolated from mouse gastrocnemius was assessed via immunoblotting to determine the levels of TAZ and mitochondrial marker proteins. Alpha-tubulin was used as a loading control. **b**) Gastrocnemius muscle of mice in panel **a** were immunostained with cytochrome c oxidase subunit 2 antibody to visualise mitochondria. Cell nuclei were counterstained with DAPI. Scale bar = 50 µm. Fluorescent signals were quantified using ImageJ software. n = 4 for each condition (**** $p < 0.0001$). **c**) Genomic DNA was isolated from gastrocnemius muscle of mice in panel **a**, and relative mitochondrial DNA copy number was determined by quantitative PCR. n = 7 for each condition (** $p = 0.0039$). **d**) TAZ was depleted by shTAZ AAV6 virus transduction in mouse gastrocnemius muscle along with AAV6-CMV virus as a control (WT+Con). At 4 days after injection, wild type or S51A mutant TAZ-expressing AAV6 virus were injected into TAZ-depleted gastrocnemius muscle to rescue TAZ (shTAZ+TAZ and shTAZ+S51A, respectively). AAV6-CMV virus was used as a control (shTAZ+Con). At 2 weeks after last injection, gastrocnemius muscle was isolated and analysed by immunoblotting to determine the level of TAZ and mitochondrial marker

proteins. Gapdh was used as a loading control. **e**) Gastrocnemius muscle of panel **d** was analysed by immunostaining with cytochrome c oxidase subunit 2 antibody. Cell nuclei were counterstained with DAPI. Scale bar = 50 μm. Fluorescent signals were quantified using ImageJ software. n = 4 for each condition (WT + Con vs. shTAZ + Con; ** $p = 0.0034$, shTAZ + Con vs. shTAZ + TAZ; * $p = 0.0287$, WT + Con vs. shTAZ + S51A; * $p = 0.034$). **f**) Genomic DNA was isolated from gastrocnemius muscle from mice in panel **d**, and relative mitochondrial DNA copy number was assessed by quantitative PCR. n = 6 for each condition (WT + Con vs. shTAZ + Con; *** $p = 0.0005$, shTAZ + Con vs. shTAZ + TAZ; ** $p = 0.0075$, WT + Con vs. shTAZ + S51A; * $p = 0.0212$). For panel **b**, **c**, **e**, and **f**, data are presented as means ± SEM. Statistical significance was analysed via two-tailed Student's *t*-test for panel **b** and **c**, or one-way ANOVA with Tukey's multiple comparison test for panel **e** and **f**. Representative data was shown and experiments were performed at least twice with similar results for panel **a** and **d**. Source data are provided as a Source Data file.

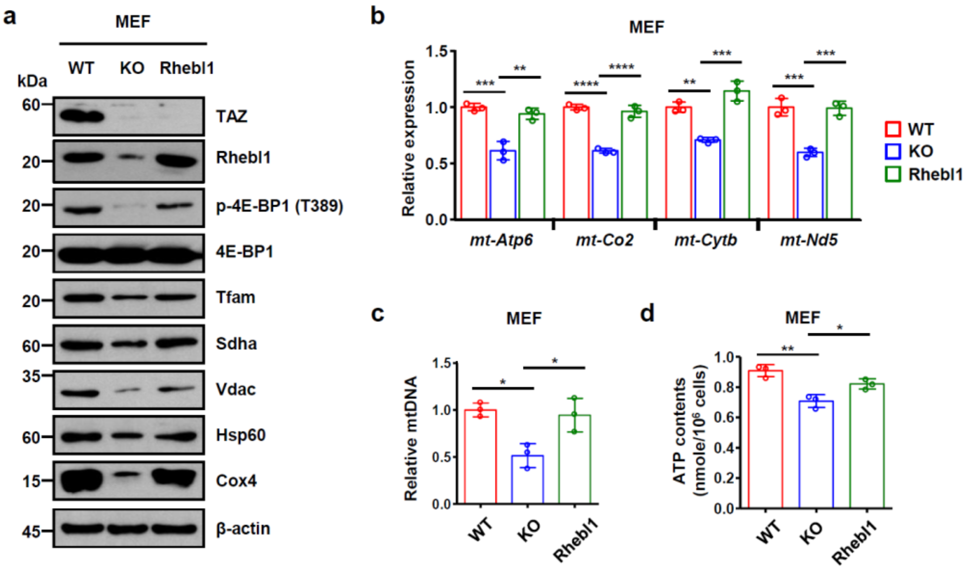

Supplementary Fig. 3. Rhebl1 rescues Tfam translation and mitochondrial biogenesis in TAZ knockout MEFs. a) Protein from wild-type (WT), TAZ knockout (KO), and Rhebl1-rescued (Rhebl1) TAZ KO mouse embryonic fibroblasts (MEF) was analysed via immunoblotting to detect the indicated proteins. Beta-actin was used as the loading control. Representative data was shown and experiment was performed twice with similar results. **b**) Quantitative reverse transcription (qRT)-PCR was performed to analyse the expression of mitochondrial marker genes of cells in panel **a**. The experiment was performed in triplicate (*mt-Atp6*; *** $p = 0.0005$ for WT vs. KO, ** $p = 0.0012$ for KO vs. Rhebl1, *mt-Co2*; **** $p < 0.0001$ for WT vs. KO, **** $p < 0.0001$ for KO vs. Rhebl1, *mt-Cytb*; ** $p = 0.0021$ for WT vs. KO, *** $p = 0.0002$ for KO vs. Rhebl1, *mt-Nd5*; *** $p = 0.0005$ for WT vs. KO, *** $p = 0.0005$ for KO vs. Rhebl1. **c**) The relative mitochondrial DNA copy number of cells in panel **a** was assessed via quantitative PCR for mitochondrial-encoded *Cox2* and nuclear-encoded *β-globin*. The Ct values of mitochondrial *Cox2* were normalized to those of nuclear *β-globin*. The experiment was performed in triplicate (WT vs. KO; * $p = 0.0103$, KO vs. Rhebl1; * $p = 0.0179$). **d**) The cellular ATP content of cells in panel **a** was analysed using the ATP bioluminescence assay. The experiment was performed in triplicate (WT vs. KO; ** $p = 0.0017$, KO vs. Rhebl1; * $p = 0.0264$). Data are presented as mean ± SD for panel **b**, **c**, and **d**. Statistical significance was analysed via one-way ANOVA with Tukey's multiple comparison test. Source data are provided as a Source Data file.

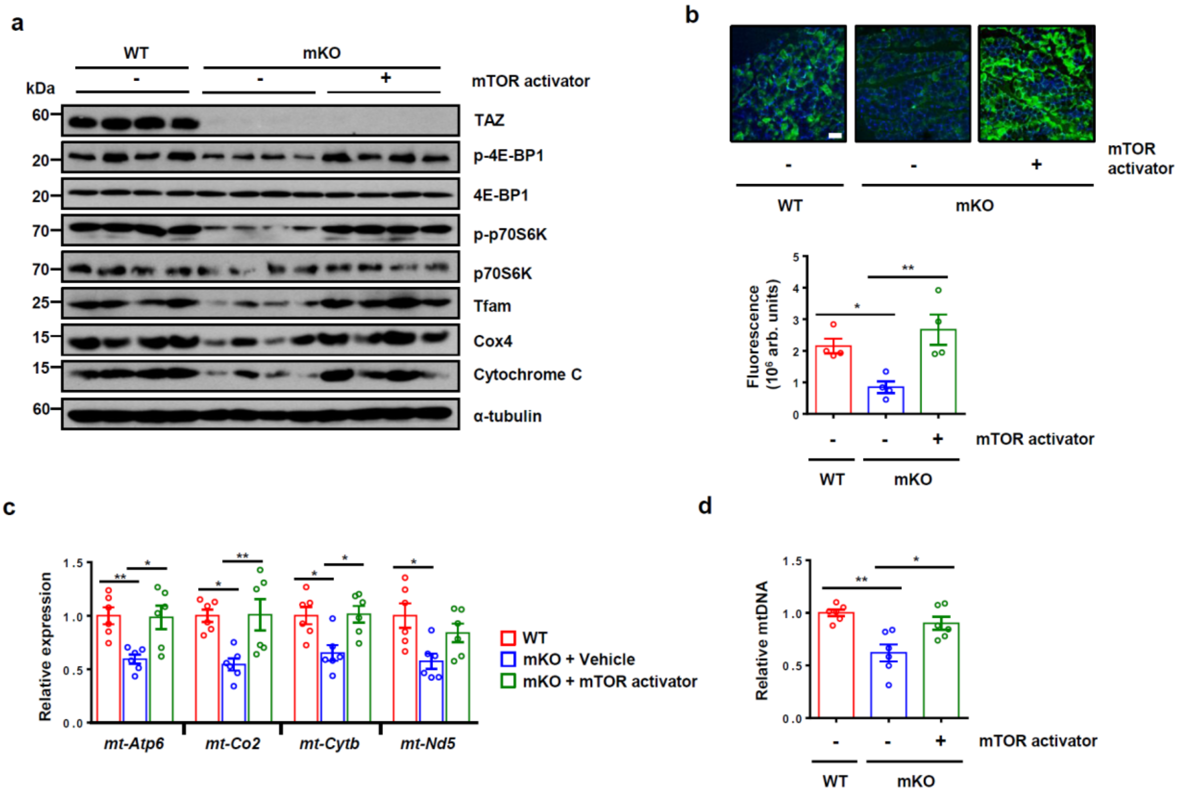

Supplementary Fig. 4. Pharmacological activation of mTOR rescued mitochondrial biogenesis in muscle-specific TAZ knockout mice. a) Protein isolated from gastrocnemius muscle of wild type (WT) and vehicle or MHY1485-administered muscle-specific TAZ knockout (mKO) mice was analysed using immunoblot assays to determine the levels of mitochondrial markers and phosphorylation status of mTOR signalling components. Alpha-tubulin was used as a loading control. Representative data was shown and experiments were performed twice with similar results. **b**) Gastrocnemius muscle of mice in panel **a** was assessed by immunofluorescent staining for Cox4 to visualise mitochondrial mass. Scale bar = 50 μm. Fluorescence was quantified via ImageJ. n = 4 for each condition (WT vs. mKO + vehicle; * *p* = 0.0461, mKO + vehicle vs. mKO + mTOR activator; ** *p* = 0.0081). **c**) RNA was isolated from gastrocnemius muscle of mice in panel **a**, and assessed by quantitative reverse transcription (qRT)-PCR to determine transcript levels of mitochondria-encoded genes. n = 6 for each condition (*mt-Atp6*; ** *p* = 0.0082 for WT vs. mKO + vehicle, * *p* = 0.0107 for mKO + vehicle vs. mKO + mTOR activator, *mt-Co2*; * *p* = 0.0112 for WT vs. mKO + vehicle, ** *p* = 0.0099 for mKO + vehicle vs. mKO + mTOR activator, *mt-Cytb*; * *p* = 0.016 for WT vs. mKO + vehicle, * *p* = 0.0123 for mKO + vehicle vs. mKO + mTOR activator, *mt-Nd5*; * *p* = 0.0137 for WT vs. mKO + vehicle). **d**) Genomic DNA derived from

gastrocnemius muscle of mice in panel **a** was analysed by quantitative PCR to quantify relative mitochondrial DNA copy number. n = 6 for each condition (WT vs. mKO + vehicle; ** $p = 0.0015$, mKO + vehicle vs. mKO + mTOR activator; * $p = 0.0146$). For panel **b**, **c**, and **d**, data are shown as means ± SEM. Statistical significance was analysed via one-way ANOVA with Tukey's multiple comparison test. Source data are provided as a Source Data file.

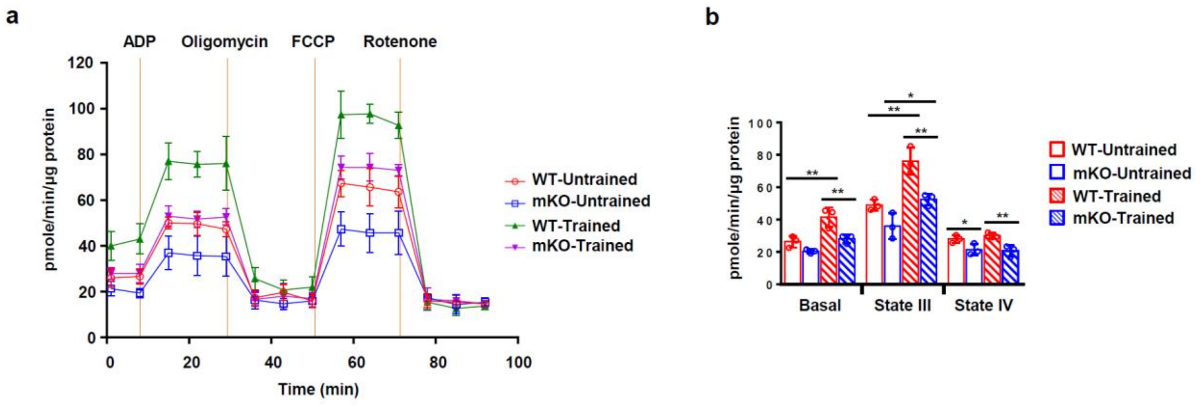

Supplementary Fig. 5. TAZ promotes exercise-induced mitochondrial respiration. a) Exercise-trained wild type (WT) and muscle-specific TAZ-knockout (mKO) mice, along with untrained WT and mKO mice, were sacrificed and live mitochondria were isolated from gastrocnemius muscle. Isolated live mitochondria were assessed by XF24 analyser to determine mitochondrial oxygen consumption rate (OCR). To measure basal, state III, and state IV respiration, 1 mM ADP, 4 μM oligomycin, 4 μM carbonyl cyanide 4-(trifluoromethoxy) phenylhydrazone (FCCP), and 4 μM antimycin A were sequentially added to mitochondria. OCR values were normalized to protein mass of isolated mitochondria. **b**) Basal, state III, and state IV respiration of single mitochondri are shown as bar graphs. For panel **a** and **b**, n = 3 for each condition (Basal; ** $p = 0.0024$ for WT-untrained vs. WT-trained, ** $p = 0.0049$ for WT-trained vs. mKO-trained, State III; ** $p = 0.0014$ for WT-untrained vs. WT-trained, * $p = 0.0249$ for mKO-untrained vs. mKO-trained, ** $p = 0.0034$ for WT-trained vs. mKO-trained, State IV; * $p = 0.0439$ for WT-untrained vs. mKO-untrained, ** $p = 0.0066$ for WT-trained vs. mKO-trained). Presented data are representative of multiple data sets. Data are shown as means ± SD. Statistical significance was analysed via two-way ANOVA with Sidak's multiple comparison test. * $P < 0.05$ and ** $P < 0.01$. Source data are provided as a Source Data file.

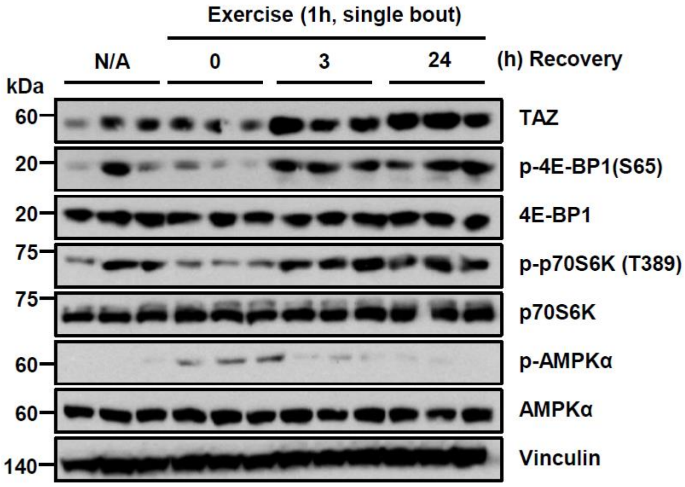

Supplementary Fig. 6. TAZ protein level and mTORC1 signaling were increased during the resting period after a single bout of exercise. Wild type mice were forced to exercise for 1 h and were euthanized 0, 3, and 24 h after resting with unexercised control mice. The level of indicated proteins from gastrocnemius muscle was assessed via immunoblotting. Vinculin was used as the loading control. Representative data was shown and experiments were performed twice with similar results. Source data are provided as a Source Data file.

Supplementary Table 1.

Primers used for gene expression analysis

gene	Direction	sequences
mt-Co2	forward	5'-GCCGACTAAATCAAGCAACA-3'
	reverse	5'-CAATGGGCATAAAGCTATGG-3'
mt-Cytb	forward	5'-CATTTATTATCGCGGCCCTA-3'
	reverse	5'-TGTTGGGTTGTTTGATCCTG-3'
mt-Atp6	forward	5'-GACGAACATGAACCCTAAT-3'
	reverse	5'-TACGGCTCCAGCTCATAGT-3'
mt-ND5	forward	5'-ACCATGCTTATCCTCACTTCAG-3'
	reverse	5'-AGTATTTGCGTCTGTTCGTCC-3'
Atp5a1	forward	5'-TGGAAACAATGTATTCGTTCAT-3'
	reverse	5'-CTCCTCCGGACGCAAAT-3'
Idh3g	forward	5'-AATGCTCAAGCCAACTCTCC-3'
	reverse	5'-TTGTTTGTTGTGAGGAAATGC-3'
Ndufa10	forward	5'-ATGCTGGTTCAGGATAAGAC-3'
	reverse	5'-TACCCTGATGAGCTCCAATA-3'
Uqcrc1	forward	5'-CACACTGCTTACCTCATCAA-3'
	reverse	5'-GACTACTGTTCTGCACGATA-3'
Tfam	forward	5'-GTCCATAGGCACCGTATTGC-3'
	reverse	5'-CCCATGCTGGAAAAACACTT-3'
Pgc1α	forward	5'-GTAGGCCCAGGTACGACAGC-3'
	reverse	5'-GCTCTTTGCGGTATTCATCCC-3'

Nrf1	forward	5'-CAACAGGGAAGAAACGGAAA-3'
	reverse	5'-GCACCACATTCTCCAAAGGT-3'
Nrf2	forward	5'-AGGTTGCCCACATTCCCAAACAAG-3'
	reverse	5'-TTGCTCCATGTCCTGCTCTATGCT-3'
Rheb	forward	5'-GGTGATGTGACAATTCTGCT-3'
	reverse	5'-GAGGTGAACTCATTTGGACA-3'
Rhebl1	forward	5'-AGGCTACGATCCTACAGTG-3'
	reverse	5'-ACAGGTTCTTAACGATCTGAAA-3'
Gapdh	forward	5'- GCTTGTCATCAACGGGAAG -3'
	reverse	5'- GATGTTAGTGGGGTCTCG -3'
**Primers for ChIP**		
Rhebl1 TBE	forward	5'- GTGTGTGAGAGAGAGAGAGAGA-3'
	reverse	5'- GATCTTCCACCTCCCAAGTG-3'
**Primers for mtDNA quantification**		
Mitochondrial *Cox2*	forward	5'-GCCGACTAAATCAAGCAACA-3'
	reverse	5'-CAATGGGCATAAAGCTATGG-3'
Nuclear *β-globin*	forward	5'-GAAGCGATTCTAGGGAGCAG-3'
	reverse	5'-GGAGCAGCGATTCTGAGTAGA-3'
**Primers for the mouse Rhebl1 promoter luciferase construct**		
Rhebl1 promoter -0.5kb	forward	5'- AAAAAAAAGCTTAAGACGAGCGGGATACCTA-3'
	reverse	5'- AAAAAACTCGAGTGGATCCCAGTGAGTTTGAAG-3'
Rhebl1 enhancer TBE	forward	5'- AAAAAAGCTAGCTCCAGGATAGCCAGAACTACA-3'
	reverse	5'- AAAAAAGGTACCGAGTCCTCTTGAAGACCAGAAAG-3'

Primers for polysome analysis		
Atp5d	forward	5'-CATGTCCCCACACTACAGGTC-3'
	reverse	5'-TCGGCATTCACAGTGACG-3'
Tfam	forward	5'-GCTTGGAAAAATCTGTCTCCTG-3'
	reverse	5'-TCGTCCAACTTCAGCCATC-3'

siRNA target sequences	
Tead4 #1	5'-ATGTGAAACCTTTCTCTCAAAACAC-3'
Tead4 #2	5'-CTGTGAGTACATGATCAACTTTATC-3'
